# Supplementary material for: Dietary fatty acid patterns and risk of oesophageal squamous cell carcinoma
Source: PeerJ. 2022 Mar 31;10:e13036. doi: 10.7717/peerj.13036 (PMC8977065; doi:10.7717/peerj.13036)
Supplement: Table S1 [file peerj-10-13036-s001.docx]

| S1 Comparison of dietary fatty acid intake between case group and control group (after energy correction) | | | | | |
| --- | --- | --- | --- | --- | --- |
| Fatty acid | controls (n=422) |  | cases (n=423) | *z* | *P* |
|  | Median(Q_25_,Q_75_) |  | Median(Q_25_,Q_75_) |  |  |
| SFA(g/day) | 11.64(8.17,15.04) |  | 12.40(8.72,18.77) | -3.270 | **0.001** |
| MUFA(g/day) | 13.32(8.89,17.21) |  | 14.49(9.69,22.17) | -3.616 | **＜0.001** |
| PUFA(g/day) | 5.04(3.50,6.88) |  | 4.91(3.34,6.45) | -4.023 | **＜0.001** |
| 4:0(mg/day) | 5.41(0.57,28.73) |  | 0.84(0.01,11.10) | -1.116 | 0.264 |
| 6:0(mg/day) | 0.96(0.02,8.51) |  | 0.03(0.01,0.99) | -6.334 | **＜0.001** |
| 8:0(mg/day) | 12.50(4.34,30.98) |  | 9.92(2.89,50.58) | -7.124 | **＜0.001** |
| 10:0(mg/day) | 20.00(7.65,48.34) |  | 11.78(2.70,28.36) | -0.110 | 0.913 |
| 11:0(mg/day) | 6.19(2.31,15.10) |  | 7.15(2.03,27.70) | -4.761 | **＜0.001** |
| 12:0(g/day) | 0.13(0.06,0.21) |  | 0.15(0.05,0.26) | -1.556 | 0.120 |
| 13:0(mg/day) | 9.07(5.56,14.33) |  | 9.77(5.75,16.92) | -1.957 | 0.050 |
| 14:0(g/day) | 0.53(0.33,0.78) |  | 0.49(0.30,0.80) | -1.490 | 0.136 |
| 15:0(g/day) | 0.18(0.08,0.33) |  | 0.14(0.05,0.26) | -0.718 | 0.473 |
| 16:0(g/day) | 7.06(4.86,9.15) |  | 7.68(5.46,11.68) | -3.871 | **＜0.001** |
| 17:0(g/day) | 0.35(0.16,0.62) |  | 0.38(0.19,0.67) | -4.155 | **＜0.001** |
| 18:0(g/day) | 2.69(1.77,3.80) |  | 3.00(2.02,4.41) | -1.184 | 0.237 |
| 19:0(g/day) | 0.031(0.01,0.047) |  | 0.023(0.01,0.039) | -3.219 | **0.001** |
| 20:0(g/day) | 0.109(0.06,0.161) |  | 0.129(0.07,0.204) | -5.688 | **＜0.001** |
| 22:0(g/day) | 0.026(0.01,0.075) |  | 0.019(0,0.078) | -3.488 | **＜0.001** |
| 24:0(mg/day) | 0.535(0.01,1.672) |  | 0.288(0,3.13) | -1.937 | 0.053 |
| 14:1(mg/day) | 3.679(1.16,10.95) |  | 1.731(0.06,6.823) | -0.141 | 0.888 |
| 15:1(mg/day) | 1.424(0.31,4.301) |  | 0.935(0.01,3.493) | -5.458 | **＜0.001** |
| 16:1(g/day) | 0.966(0.57,1.306) |  | 1.099(0.68,1.566) | -2.515 | **0.012** |
| 17:1(g/day) | 0.066(0.02,0.158) |  | 0.043(0.01,0.103) | -3.543 | **＜0.001** |
| 18:1(g/day) | 12.07(8.01,15.6) |  | 13.33(8.92,20.16) | -4.961 | **＜0.001** |
| 20:1(g/day) | 0.03(0.02,0.047) |  | 0.039(0.02,0.06) | -4.239 | **＜0.001** |
| 22:1(g/day) | 0.042(0.02,0.093) |  | 0.039(0.01,0.086) | -5.398 | **＜0.001** |
| 24:1(mg/day) | 0.866(0.01,4.878) |  | 0.323(0.01,8.735) | -1.718 | 0.086 |
| 16:2(g/day) | 0.032(0.01,0.063) |  | 0.04(0.01,0.073) | -0.100 | 0.920 |
| 18:2n-6(g/day) | 4.199(2.85,5.519) |  | 3.864(2.72,5.203) | -2.144 | **0.032** |
| 18:3n-3(g/day) | 0.534(0.36,0.852) |  | 0.423(0.26,0.708) | -1.488 | 0.137 |
| 18:4n-3(mg/day) | 0.499(0.47,0.516) |  | 0.514(0.49,0.525) | -5.077 | **＜0.001** |
| 20:2n-6(mg/day) | 59.42(30.2,98.37) |  | 74.31(35.2,132.6) | -7.446 | **＜0.001** |
| 20:3n-3(mg/day) | 2.452(0.88,6.51) |  | 2.178(0.29,6.89) | -3.702 | **＜0.001** |
| 20:4n-6(mg/day) | 55.83(33,88.67) |  | 64.13(40.1,97.87) | -1.346 | 0.178 |
| 20:5n-3(mg/day) | 29.95(5.47,92.67) |  | 34.63(1.85,138.3) | -2.791 | **0.005** |
| 22:3n-3(mg/day) | 0.175(0,2.185) |  | 0.008(0,2.808) | -0.470 | 0.638 |
| 22:4n-6(mg/day) | 6.325(2.22,14.22) |  | 4.859(1.41,15.12) | -0.957 | 0.338 |
| 22:5n-3(mg/day) | 0.816(0.1,2.982) |  | 0.203(0.01,4.151) | -2.022 | **0.043** |
| 22:6n-3(mg/day) | 0.05(0.01.0.15) |  | 0.04(0.01,0.23) | -3.418 | **0.001** |
