# Supplementary material for: Dietary fatty acid patterns and risk of oesophageal squamous cell carcinoma
Source: PeerJ. 2022 Mar 31;10:e13036. doi: 10.7717/peerj.13036 (PMC8977065; doi:10.7717/peerj.13036)
Supplement: Table S2 — 1 Spearman’s correlation coefficients; * all p < 0.05 [file peerj-10-13036-s002.docx]

S2 Correlation between dietary fatty acid scores and food groups

|  |  | cereals | meat | freshwater fish | deep-sea fish | peanut oil | animal oil | blend oil |
| --- | --- | --- | --- | --- | --- | --- | --- | --- |
| Pattern1 | r^1^ | -0.04 | 0.02 | -0.077 | 0.074 | 0.04 | 0.124 | -0.086 |
|  | *P* | 0.326 | 0.618 | 0.06 | 0.07 | 0.303 | 0.002* | 0.03* |
| Pattern2 | r^1^ | -0.055 | 0.063 | 0.031 | 0.178 | 0.189 | -0.008 | -0.131 |
|  | *P* | 0.177 | 0.125 | 0.455 | ＜0.001* | ＜0.001* | 0.852 | 0.001* |
| Pattern3 | r^1^ | 0.052 | 0.017 | 0.006 | 0.039 | 0.038 | 0.022 | -0.041 |
|  | *P* | 0.208 | 0.675 | 0.876 | 0.339 | 0.331 | 0.581 | 0.305 |
| Pattern4 | r^1^ | 0.021 | 0.021 | 0.006 | 0.099 | 0.046 | -0.013 | 0.048 |
|  | *P* | 0.615 | 0.615 | 0.881 | 0.015* | 0.235 | 0.756 | 0.224 |

^1^ Spearman’s correlation coefficients; * all p < 0.05
