# Supplementary material for: Dietary fatty acid patterns and risk of oesophageal squamous cell carcinoma
Source: PeerJ. 2022 Mar 31;10:e13036. doi: 10.7717/peerj.13036 (PMC8977065; doi:10.7717/peerj.13036)
Supplement: Table S3 — * all p < 0.05 [file peerj-10-13036-s003.docx]

S3 Correlation between dietary fatty acid patterns and clinical pathological factor

|  |  | T | N | M |
| --- | --- | --- | --- | --- |
| MLC-SFA | r_s_ | -0.002 | 0.117 | 0.093 |
|  | *P* | 0.969 | 0.052 | 0.121 |
| EC-UFA | r_s_ | 0.076 | 0.270 | 0.061 |
|  | *P* | 0.219 | <0.001* | 0.310 |
| SFA | r_s_ | 0.006 | -0.057 | 0.108 |
|  | *P* | 0.925 | 0.340 | 0.070 |
| n-3 LC-PUFA | r_s_ | -0.112 | 0.036 | -0.175 |
|  | *P* | 0.070 | 0.548 | 0.003* |

* all p < 0.05
